# The Relationship between Vascular Biomarkers (Serum Endocan and Endothelin-1), NT-proBNP, and Renal Function in Chronic Kidney Disease, IgA Nephropathy: A Cross-Sectional Study

**DOI:** 10.3390/ijms251910552

**Published:** 2024-09-30

**Authors:** Balázs Sági, Tibor Vas, Csenge Gál, Zoltán Horváth-Szalai, Tamás Kőszegi, Judit Nagy, Botond Csiky, Tibor József Kovács

**Affiliations:** 1 2nd Department of Internal Medicine and Nephrology, Diabetes Center, Clinical Centre, Medical School, University of Pécs, 7624 Pécs, Hungary; vas.tibor@pte.hu (T.V.); judit.nagy@aok.pte.hu (J.N.); botond.csiky@gmail.com (B.C.); kovacs.tibor.jozsef@pte.hu (T.J.K.); 2 National Dialysis Center Pécs, 7624 Pécs, Hungary; 3 Department of Laboratory Medicine, Medical School, University of Pécs, 7624 Pécs, Hungary; gal.csenge@pte.hu (C.G.); horvath-szalai.zoltan@pte.hu (Z.H.-S.); koszegi.tamas@pte.hu (T.K.); 4 Molecular Medicine Research Group, Szentágothai Research Center, University of Pécs, 7624 Pécs, Hungary

**Keywords:** chronic kidney disease, IgA nephropathy, endocan, endothelin-1, renal function, NT-proBNP

## Abstract

IgA nephropathy (IgAN) is the most common primary glomerular disease. Endothelin-1 (ET-1) is one of the strongest vasoconstrictor materials in the blood. The N-terminal prohormone of brain natriuretic peptide (NT-proBNP) is associated with renal function and poor outcomes in chronic kidney disease (CKD). Serum endocan is a biomarker associated with proinflammatory cytokines, and the increase in the serum level plays a critical role in inflammatory, proliferative, and neovascularization processes and is associated with poor cardiovascular outcomes in patients with CKD too. Identifying high-risk patients using biomarkers could help to optimize their treatment. Ninety patients with biopsy-confirmed IgAN were included in the study (50 males/40 females, mean age: 54.9 ± 14.4 years). Serum endocan, ET-1, and NT-proBNP were measured by enzyme-linked immunosorbent assay kits. Echocardiography was performed, and carotid-femoral pulse wave velocity (cfPWV) was measured by SphygmoCor in this cross-sectional study. Patients were divided into two groups based on serum endocan median level (cut-off: 44 ug/L). There was significantly higher aorta systolic blood pressure (SBPao) (*p* = 0.013), NT-proBNP (*p* = 0.028), albumin/creatinine ratio (*p* = 0.036), and uric acid (*p* = 0.045) in the case of the higher endocan group compared to the lower. There was also significantly higher SBPao (*p* = 0.037) and NT-proBNP (*p* = 0.038) in the case of higher endothelin-1 (ET-1) levels compared to the lower (cut-off: 231 pg/mL) group by the two-sample *t*-test. Then, we divided the patients into two groups based on the eGFR (CKD 1–2 vs. CKD 3–5). The levels of serum endocan, NT-proBNP, cfPWV, SBPao, left ventricular mass index (LVMI), uric acid, and albuminuria were significantly higher in the CKD 3–5 group compared to the CKD 1–2 group. The serum endocan and NT-proBNP levels were significantly higher in the diastolic dysfunction group (*p* = 0.047, *p* = 0.015). There was a significant increase in serum endocan levels (CKD 1 vs. CKD 5; *p* = 0.008) with decreasing renal function. In IgAN, vascular biomarkers (endocan, ET-1) may play a role in endothelial dysfunction through vascular damage and elevation of SBPao. Serum endocan, ET-1, and NT-proBNP biomarkers may help to identify IgAN patients at high risk.

## 1. Introduction

Chronic kidney disease (CKD) poses a worldwide health challenge for health professionals and healthcare providers globally [1,2]. IgA nephropathy (IgAN) is the most common primary glomerular disease and the typical CKD for cardiovascular (CV) risk assessment. IgAN is caused by the forming of abnormal IgA molecules and their deposition in the kidney glomeruli, leading to a series of inflammatory reactions that ultimately lead to irreversible glomerulosclerosis. The kidney function deteriorates to end-stage renal failure (ESRF) in 25–50% of IgAN patients, which will then be responsible for a decreased quality of life, the necessity for dialysis or transplantation, and a risk of early mortality [3].

Elevated serum creatinine levels at first presentation; persistent hypertension; proteinuria; and specific histological features such as mesangial hypercellularity, segmental glomerulosclerosis, tubular atrophy, or interstitial fibrosis are well-known risk factors for poor clinical outcomes in IgAN [4,5]. However, none of these markers are sensitive or specific; therefore, their prediction of the risk of disease progression continues to be disputed.

Several studies have described modern kidney biomarkers that might anticipate renal outcome independently, but these markers ought to be approved more altogether [6,7,8].

Serum endocan is known as a dermatan sulfate and a mature polypeptide of 165 amino acids, making up the 50-kDa proteoglycan, an endothelial-cell-specific molecule-1 [9]. Endocan is a soluble molecule that is secreted from vascular endothelial cells of various organs and can freely circulate in the blood [10], in contrast to other ubiquitous proteoglycans, which are mostly found in connective tissue. It is still unclear what endocan does in humans. In any case, it has been accounted for that raised plasma endocan levels could act as an autonomous gamble factor for unfortunate endurance in patients with harm, ongoing kidney disease (CKD), toxemia, sepsis, and hypertension [11,12,13,14,15]. Observational data show that endocan also plays a key role in the occurrence and progression of systemic lupus erythematosus, psoriasis, and type 2 diabetes. The presence of these conditions will lead to a higher likelihood of cardiovascular events, but it is unclear how endocan contributes to the development of these conditions. Endocan is a non-specific material, which reflects chronic vascular cell injury. Therefore, Chen et al.’s review suggests endocan is not enough to be used as a biomarker alone and should be used together with other disease biomarkers [16].

A few examinations have reported endothelial injury in patients with IgAN [17,18]. Recent research shows that endocan may indicate all-cause mortality and cardiovascular events in patients with chronic kidney disease as well [12,16], but it was not examined in the IgAN subgroup.

It could be used additionally as a prognostic factor of the unfavorable cardiovascular outcome, especially in patients on peritoneal dialysis displaying rapid decline of urine output volume [19,20]. The importance of endocan level rise in IgAN and decline of renal function of IgAN is unknown. Still, elevated plasma concentrations of endocan may serve as an independent predictor for the rapid decline of renal function in IgA nephropathy but based on these presumably in a non-specific manner.

In the meta-analysis of Khalaji et al., they conclude that endocan showed promising impress in predicting cardiorenal complications among CKD patients [21].

The involvement of endothelin-1 (ET-1) in the advancement of chronic kidney disease and the onset of hypertension is also fairly well understood [22,23]. ET-1 is a type of peptide hormone that is created and secreted by endothelial cells. It raises blood pressure because it is also a potent vasoconstrictor. It might be involved in cardiovascular diseases like high blood pressure, heart attacks, and strokes. ET-1 plays an important role in cell migration, cell division, regulation of cell division, regulation of cell functions, and maintenance of cell composition. However, its effects seem to be sensitive to changes in the extracellular environment and the physiological state of the cells [24,25,26]. Multiple animal and human models have demonstrated that the activation of endothelin A receptors by endothelin-1 (ET-1) contributes to cell injury, proteinuria, inflammation, and fibrosis in CKD [26].

ET-1 has multiple effects in the kidney, in many pathways, and it has a prominent vasoconstrictive effect in the kidney vasculature. In the glomerulus, it can cause podocytopathies, and it produces reactive oxygen species and also acts in the tubulointerstitial compartment, as well as having interactions and crosstalk between the RAAS system [27].

Recently it was found that sparsentan, which is a dual endothelin and angiotensin II receptor antagonist, could reduce proteinuria and kidney function loss in cases of high rapid decline renal function IgAN patients, but further results are pending [28].

Natriuretic peptides (such as NT-proBNP) are independently associated with prevalent cardiovascular disease (CVD) and future CV events in cross-sectional [29,30] and prospective [31,32,33,34] CKD cohort studies. In our previous study, we found an association between NT-proBNP, carboxy-terminal telopeptide of collagen type I (CITP) biomarkers, and renal function in IgAN patients [35].

In a recent study by Yoon et al., they identified 3 urinary biomarkers from 16, which was correlated with the progression of IgAN; only urinary growth/differentiation factor-15 (GDF-15), interleukin-6 (IL-6), and epidermal growth factor (EGF) were associated with poor renal outcome, but urinary endocan was not [36].

But despite all these previous clinical data, we have little information about the correlations in the case of combined biomarker tests to identify high-risk IgAN patients with CV disease and heart failure. Cardiovascular diseases are the leading cause of mortality in CKD as a result of cardiovascular diseases caused by deteriorating kidney function and many other risk factors [37].

We hypothesized that endocan, ET-1, and NT-proBNP may play a role in endothelial dysfunction in IgAN patients because endothelial injury could be a key factor in the progression of CKD [38], and we should use them as new biomarkers for progressive cases. The point of this study was to assess if there is any relationship between plasma endocan, endothelin-1, and NT-proBNP levels with the deteriorating renal function in patients with IgAN.

## 2. Results

**Figure 1:**
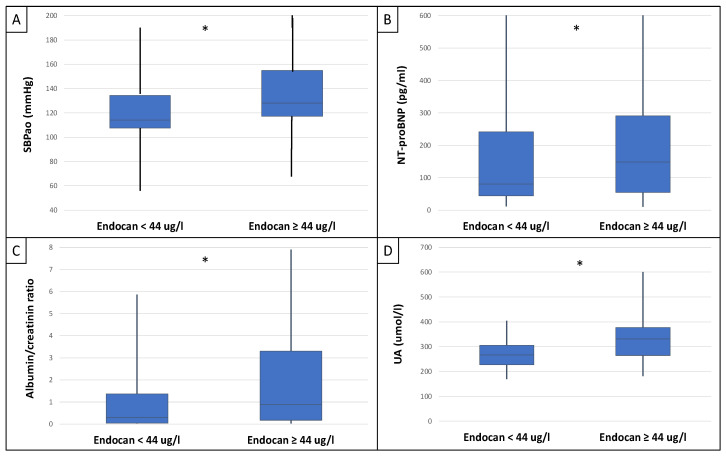
Differences in SBPao (**A**), NT-proBNP (**B**), albumin/creatinine ratio (**C**), and uric acid level (**D**) in the cases of low and high endocan level groups (cut-off: 44 ug/L) in IgAN patients. Statistical analysis was performed by Student’s *t*-test. (* *p* < 0.05).

**Figure 2:**
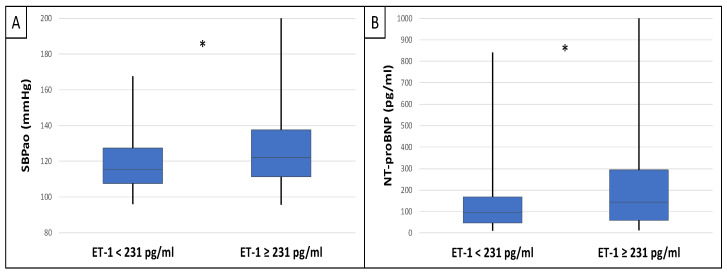
Differences in SBPao (**A**) and NT-proBNP (**B**) in the case of low and high endothelin-1 level groups (cut-off: 231 pg/mL) in IgAN patients. Statistical analysis was performed by Student’s *t*-test (* *p* < 0.05).

**Figure 3:**
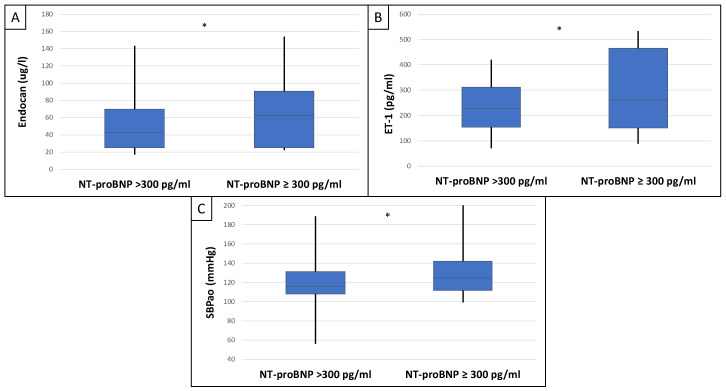
Differences in endocan (**A**), endothelin-1 (**B**), and SBPao (**C**) in the case of low and high NT-proBNP level groups (cut-off: 300 pg/mL) in IgAN patients. Statistical analysis was performed by Student’s *t*-test (* *p* < 0.05).

**Figure 4:**
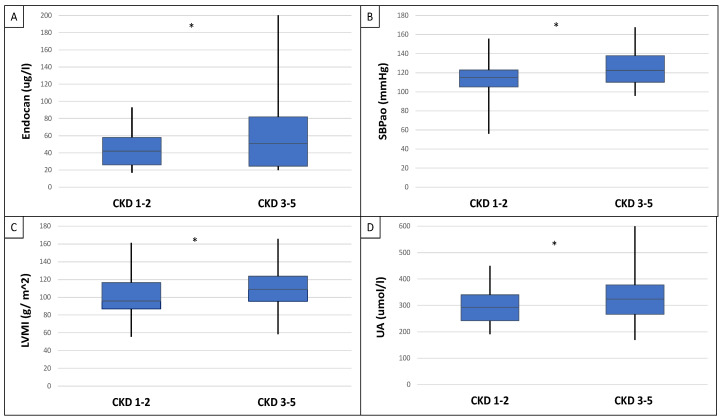
Differences in serum endocan level (**A**), SBPao (**B**), LVMI (**C**), and uric acid (**D**) between CKD 1–2 vs. CKD 3–5 groups of IgAN patients. Statistical analysis was performed by Student’s *t*-test (* *p* < 0.05).

**Figure 5:**
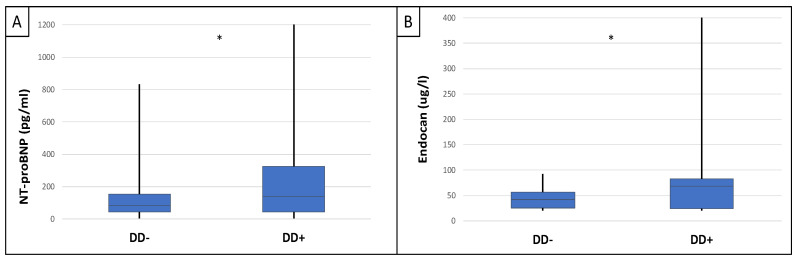
Differences in serum NT-proBNP (**A**) and endocan (**B**) levels between IgAN patient groups with and without diastolic dysfunction. Statistical analysis was performed by Student’s *t*-test (* *p* < 0.05).

**Figure 6:**
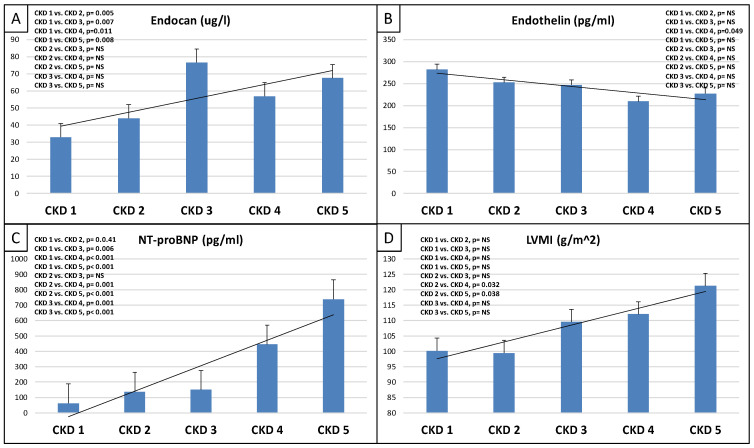
Tendencies in changes of serum endocan level (**A**), serum endothelin level (**B**), NT-proBNP (**C**), and LVMI (**D**) from CKD 1 to CKD 5. NT-proBNP: N-terminal prohormone of brain natriuretic peptide; CKD: chronic kidney disease; LVMI: left ventricular mass index. NS: not significant. Statistical analysis was performed by Student’s *t*-test and Jonckheeres trend test analysis.

This study included 90 patients, 50 of whom were male and had an average age of 54.9 ± 14.4 years. Table 1 displays the clinical characteristics of the patient. Patients were divided into two groups based on serum endocan median level (cut-off: 44 ug/L). There was significantly higher aorta systolic blood pressure (SBPao) (*p* = 0.013), NT-proBNP (*p* = 0.028), albumin/creatinine ratio (*p* = 0.036), uric acid (*p* = 0.045), and relative wall thickness (RWT) (*p* = 0.026) in the case of the higher endocan group compared to the lower (Figure 1). There was also significantly higher SBPao (*p* = 0.037), NT-proBNP (*p* = 0.038), and mean arterial pressure (MAP) (*p* = 0.018) in the group with higher endothelin-1 (ET-1) levels compared to the lower (cut-off: 231 pg/mL), but there was no significant difference in the systolic and diastolic blood pressure between the groups (Figure 2). Then, we divided the patients into two groups based on the NT-proBNP level (cut-off: 300 pg/mL) and there were significantly higher endocan (*p* = 0.037), ET-1 (*p* < 0.001), and SBPao (*p* = 0.019) levels in case of the higher NT-proBNP group compared to the lower group (Figure 3). We formed two other groups based on the eGFR (CKD 1–2 vs. CKD 3–5). The serum endocan (*p* = 0.047), SBPao (*p* = 0.011), left ventricular mass index (LVMI) (*p* = 0.022), and uric acid (*p* = 0.027) were significantly higher in the CKD 3–5 group compared to the CKD 1–2 group (Figure 4). The serum endocan and NT-proBNP levels were significantly higher in the diastolic dysfunction group (*p* = 0.047, *p* = 0.015) (Figure 5). There was a significant increasing tendency in serum endocan level (CKD 1 vs. CKD 5; *p* = 0.008), NT-proBNP (CKD 1 vs. CKD 5; *p* < 0.001), LVMI (CKD 2 vs. CKD 5; *p* = 0.038), and SBPao (CKD 1 vs. CKD 5; *p* = 0.040) with the decreasing renal function (Figure 6). Endocan showed a positive Spearman’s correlation with the SBPao (r = 0.253, *p* = 0.022), and the endothelin-1 also showed a positive correlation with HDL-cholesterol (r = 0.259, *p* = 0.017) (Table S1). Multivariate regression analysis showed a significant association between endocan and body mass index (B: −4.299, CI: −7.842–−0.756, *p* = 0.018), as well as between ET-1 and age (B: −2.420, CI: −4.579–−0.262, *p* = 0.029) (Table S2). 

## 3. Discussion

In the present study, we demonstrated that increased serum endocan and endothelin-1 are associated with deteriorating renal function in IgAN. Our former examination demonstrated that NT-proBNP may predict some cardiac complications in IgAN [35]. In our present study, the central BP (SBPao) was higher in the higher endocan and endothelin-1 group, and it is well known that higher BP indicates worse progression in CKD. Based on our results, the endocan and endothelin-1 as vascular biomarkers and NT-proBNP may help to identify IgAN patients at high risk for subclinical heart failure and further atherosclerotic disease.

Endocan is primarily produced by endothelial cells in the blood vessels of the kidney and lung [38]. Its secretion by the activated endothelium and its regulatory effect in inflammation and endothelial dysfunction could explain the significant increase in plasma levels in several types of kidney and other diseases, such as type 2 diabetes, psoriasis, and systemic lupus erythematosus. It is not known whether the endocan levels in homogenous chronic kidney disease are as IgAN.

Samouilidou et al.’s recent review reveals that endocan could be a predictor of all-cause mortality and cardiovascular events in chronic kidney disease patients [39]. It could be used as a prognostic factor of the unfavorable cardiovascular outcome, especially in patients on peritoneal dialysis displaying a rapid decline of urine output volume. On the other hand, endocan levels could efficiently contribute to the diagnosis of several kidney disorders, such as acute kidney injury secondary to inflammation and endothelial damage [40]. Based on our results, endocan may play a role not only in acute cases but also in chronic processes because its level increases.

In a cross-sectional study, Xu et al. [41] compared plasma endocan levels of 80 patients with hyperuricemic nephropathy in different stages of chronic kidney disease (CKD). They found higher levels of endothelial markers, including endocan, in CKD stages 3–4 compared to those in stages 1–2. Similar findings were observed in our study in the endocan level of IgAN patients. Other research also demonstrated higher endocan concentrations in hemodialysis (HD) patients compared to those in the end stages of CKD and healthy individuals [42], indicating a correlation between CKD stage and endocan levels.

In Lee et al.’s study, they investigated the clinical relevance of plasma and urine endocan levels in 64 patients with IgAN and found that an elevated plasma endocan level posed an independent risk for the rapid decline of kidney function [43]. However, they did not find any differences in the level of serum endocan.

Also in Lee’s study, both plasma and urine endocan levels were found to be markedly elevated in individuals with IgAN compared to those in good health, according to their findings. Plasma and urine endocan levels were elevated in patients with advanced pathologic grades when classified using the Lee grading system. In contrast, there was no significant correlation between Oxford classification variables and plasma and urine endocan levels in their study [43]. We did not examine the histological alterations in association with the endocan. Lee et al. also investigated urine endocan levels in IgAN.

The basement membrane in a healthy glomerulus typically carries a negative charge, which helps to block the passage of molecules around 50–60 kDa molecular weight, which have negative charges (e.g., albumin). The endocan is due to the presence of dermatan sulfate, a key negatively charged component of endocan [9]. Therefore, in healthy people, there is no detectable endocan level in the urine, which was found in the controls of Lee’s study. On the contrary, any already mild damage to the glomerular basement membrane could result in the leakage of circulating plasma endocan into the urine. On the other hand, endocan might also escape into the urine through the renal tubules. Indeed, immunohistochemistry staining in healthy control biopsies showed endocan expression in the renal tubular epithelium [10]. However, most IgAN patients had different levels of urine endocan in Lee’s study. These findings implied that endocan was involved in important pathophysiologic processes in the kidneys of IgAN patients [43]. In our study, we did not examine the patient’s urine endocan level. In the aforementioned study, urine endocan level was not associated with renal outcomes. However, Lee et al. determined that the rise in plasma endocan levels could be a valuable predictor of negative kidney outcomes in IgAN patients, as it could indicate vascular endothelial damage related to disease development and CKD advancement, as opposed to the rise in endocan levels in urine, which may be caused by glomerular membrane injury [43]. Our study found significant differences in different CKD stages and an increasing tendency in serum endocan levels with deteriorating renal function. This may also support the importance of the pathomechanism mentioned above (Figure 6).

Some previous studies supported the hypothesis that endothelial dysfunction contributed to IgAN pathogenesis and CKD progression [16,17]. Yilmaz et al. reported a positive correlation between plasma endocan levels and CKD stages [12]. They proposed that plasma endocan could be an indicator of both mortality and cardiovascular events. Our study was only cross-sectional, but further follow-up of our patients may confirm this observation.

Past research has shown contradictory findings regarding the biological functions of plasma endocan in regulating inflammation. Bechard et al. [44] showed that endocan could bind directly to lymphocyte-function-associated antigen-1 (LFA-1), a major integrin in human monocytes and lymphocytes. The interaction between leukocyte LFA-1 and vascular endothelial intercellular adhesion molecule-1 is a pivotal process in leukocyte adhesion and migration. Thus, endocan binding to LFA-1 suggested a mechanism for the anti-inflammatory effect of endocan. The question of whether endocan has a proinflammatory or anti-inflammatory role in vivo still needs to be addressed. We detected differences in plasma endocan levels among different CKD stages. Based on these, the increasing endocan level in parallel with the severity of CKD that we experienced confirms the data that described increased endothelial damage and oxidative stress in the uremic state.

Arman et al. [45] investigated the effects of glycemic regulation on serum endocan levels in patients with diabetes and found that the degree of glycemic control could influence serum endocan levels. Thus, they presume that high plasma endocan levels in patients with advanced CKD might result from poor glycemic control, rather than the CKD stage. In future studies, the degree of glycemic control (i.e., hemoglobin A1c) should be assessed to investigate this possibility. Only 16 (18%) of our patients had diabetes, so based on these, it can be assumed that not only poor glycemic control but also a more serious kidney condition (uremic condition) may be responsible for the rising endocan level.

We found an association between eGFR and endocan, endothelin-1, and NT-proBNP in IgA nephropathy. In cases of deteriorated renal function, there was a significantly higher aorta systolic blood pressure, which is a well-known process. In addition to endocan, as CKD progresses, NT-proBNP also increases, but the parallel ET-1 determination did not show such an increase in the ET-1 level. The ET-1 level was significantly higher in the CKD1–2 group than in the CKD 3–5 group (Figure 4).

Our knowledge of the mechanisms underlying the advancement of kidney disease, cardiovascular disease, and all-cause mortality has greatly benefited from measurements of arterial stiffness [46], which was supported by our former examinations as well [47,48], but in this current study, we did not find any association between PWV and endocan and endothelin-1. In the case of NT-proBNP and eGFR, we found an association with PWV similar to our former study [35].

Ohno et al. found that in CKD patients, central blood pressure is a stronger predictor of CV and renal disease outcomes compared with brachial blood pressure and should therefore be used to guide antihypertensive therapy [49]. Our results supported these observations because only central BP was significantly higher among the patients with elevated endocan and ET-1 levels, but the value of the causal measurements was not, although it should be taken into account that the number of cases was not high in our study.

Also in our previous and present research, we came to the same conclusion as Bansal et al., who identified NT-proBNP as a highly effective indicator of CV prognosis in CKD IgAN patients not receiving dialysis [34,35]. They demonstrated that, even after complete adjustment for potential confounders, NT-proBNP remains a robust predictor of outcome, notably for eGFR in IgAN. This finding is important because previous claims [30,50] suggested that the independence of using this plasma biomarker as a CV outcome marker in CKD might be hindered by the strong link between NT-proBNP and GFR in advanced CKD due to reduced renal clearance of NT-proBNP.

Serum ET-1 plays a role in the physiology of vascular homeostasis, ageing, and cellular senescence. More diseases (hypertension, diabetes, obesity, CKD, heart failure) are triggers of ET-1 overproduction, and ET-1 is also responsible for disease development and progression. The main key point could be vascular inflammation caused by ET-1 overproduction [51].

In our study, we found that the ET-1 level was lower in the higher endocan level group compared to the lower endocan level group of IgAN patients, but the NT-proBNP was significantly higher. In the higher ET-1 level group, there was also a higher NT-proBNP level, but the endocan level difference between the higher and lower ET-1 level group was not significant. In the case of the CKD group comparison (CKD 1–2 vs. CKD 3–5), there were no significant differences in endocan and endothelin levels. These results suggest speculation that not only the renal function deterioration causes the biomarker elevation but also that impaired vascular remodeling and inflammation caused by elevated central aortic blood pressure could be one of these factors.

While there was no significant difference between the groups separated by the endocan cut-off, diabetes and hypertension are serious influencing factors for the course of the disease in IgAN, and we believe that they have also affected the biomarker levels. It is somewhat unexpected that the rates of diabetes and hypertension were substantially lower in the other groups split according to the endothelin-1 cut-off. It should come as no surprise that we also discovered noticeably higher rates of diabetes and hypertension in CKD 1–2 compared to CKD 3–5. We believe that a subgroup study (with and without DM and HT) would yield more accurate results; nevertheless, the number of patients in our group was modest. A follow-up study to identify the applicability of these biomarkers would be useful.

Previous examinations showed that high uric acid level is associated with faster progression and worsening proteinuria and renal outcomes in IgAN patients [52,53]. In our study, we also found a higher uric acid level in the higher endocan level group, and therefore we think that the explanation for this could be the increased endothelial injury in the higher endocan level group IgAN patients.

Wu et al.’s study described a positive correlation between serum endocan and cfPWV in ESKD patients on maintenance hemodialysis [54]. We found in the same alteration between CKD 1–2 vs. CKD 3–5 that not only did the cfPWV differ significantly from the groups but also the SBPao was significantly higher in the lower eGFR group. All of this highlights that the SBPao measurement could help clinicians with further risk stratification in IgAN.

In the vasculature, the first sign of vascular abnormalities could be endothelial dysfunction, which results in elevated serum endocan and endothelin-1 levels. In the heart, the sign of myocardial remodeling will be the NT-proBNP level increase. Peng et al. discovered significant correlations between declining kidney function and the left ventricular mass index (LVMI) and found that LVMI was closely linked to ET-1 in patients with CKD [55]. In our study results, we observed a significant difference only in the relative wall thickness (RWT) between the higher ET-1 group and the lower group, as well as a significantly higher LVMI in the CKD 3–5 group. We may propose that there is a single pathomechanism underlying the aberrant vascular and myocardial remodeling in IgAN patients, which would corroborate the biomarker data and the outcome that we could identify by echocardiography (LVH and DD). These patients should be regarded as high-risk individuals, and it is likely that they will have higher biomarker levels.

In response to the compounding effects of traditional risk factors and CKD-related risk factors, these structural changes together predispose to CV events and heart failure. However vascular remodeling and cardiomyocyte hypertrophy may represent adaptive reactions to pressure and volume stress [56]. So, maybe we can partially predict the development of complications in CKD, particularly cardiovascular issues, to decrease mortality and enhance the survival and quality of life in CKD patients.

## 4. Materials and Methods

Our cross-sectional study involved 90 patients with confirmed IgAN based on renal biopsies, and we conducted a retrospective analysis of their data. The University of Pécs Regional Research Ethics Committee approved the study protocol, and all participants provided written consent to its completion. The inclusion criterion for the study was histologically confirmed IgAN over 18, free from any symptoms and signs of heart failure, or being diagnosed heart failure. Exclusion criteria were previous or current immunosuppressive treatment (due to the modifying effects of the biomarkers) and severe comorbidities (such as malignancies that required active treatment or acute infection, severe hepatic impairment, active autoimmune diseases) or uncontrolled blood pressure (over 160 mmHg). All of them were free from renal replacement therapy.

At the start of the patient enrolment, echocardiography measurements were performed, and classic CV risk factors, including hypertension, carbohydrate metabolism disorder, obesity, lipid abnormalities, smoking, and patient medication for treating high blood pressure (ACEI/ARB, BB, CCB) and statins, were also documented. The obesity inclusion criterion was a BMI over 30 kg/m^2^. The CKD-EPI formula was used to estimate renal function (eGFR, mL/min, 1.73 m^2^). Patients who had severe comorbidities (malignancies that required active treatment) and were on corticosteroids were excluded. A 24 h blood pressure monitor was used by Meditech ABPM devices to determine the patient’s 24 h average systolic and diastolic blood pressure, pulse pressure, and diurnal index.

### 4.1. Biomarker Measurement

N-terminal fragment of brain natriuretic peptide (NT-proBNP) was determined in the accredited Department of Laboratory Medicine (University of Pécs, NAH-9-0008/2021) by a fully automated immunoassay method (Roche^®^ GmbH, Mannheim, Germany). The automated assay was calibrated by a master-type method using two calibrators included in the reagent kit. The measuring range was 36–35,000 pg/mL with an intraassay precision at two various concentrations of 2.5% and 3.0% coefficient of variation, respectively. The interassay precision was also provided at two levels of NT-proBNP samples and were found to be 4.5% and 4.8% CV, respectively. Serum endocan and endothelin-1 (ET-1) were measured using enzyme-linked immunosorbent assay (ELISA) kits (Endocan and endothelin-1 kits by MyBioSource^®,^ San Diego, CA, USA) in the Szentágothai Research Center (University of Pécs), following the manufacturer’s protocol. Endocan was a sandwich-type assay with an 8-point calibration in the range of 0–10 µg/L. The manufacturer’s data showed an intraassay precision of CV < 10%, while the interassay precision was given as CV < 12%, both determined at three endocan concentration levels. Serum endothelin-1 was measured by a competitive assay using 6 calibrators in the range of 0–500 pg/mL. The three-level intraassay precision was given as CV < 10% with an interassay precision of the method at CV < 12%.

### 4.2. Arterial Stiffness Measurement

Arterial stiffness was determined by measuring carotid-femoral pulse wave velocity (cfPWV). The measurement of cfPWV was conducted using applanation tonometry with the SphygmoCor System from AtCor Medical in Sydney, Australia. Measurements were performed in the morning in the supine position after at least 10 min of rest in a quiet, temperature-controlled room. The recording of pulse waves was conducted in sequence at two superficial artery locations (carotid-femoral segment). The cfPWV was calculated. Also, central aortic pressure was measured by the device. The augmentation index (Aix) is based on blood pulse-wave reflection and is an accepted measure of arterial stiffness and risk factors for cardiovascular disease. The Aix is widely recognized as a way to measure the increase in central aortic pressure caused by a reflected pulse wave, as we described in our previous study [35].

### 4.3. Echocardiographic Measurement

Echocardiography was performed with the Aloka SSD 1400. The left ventricular mass (LVM) was derived by using 2D images of the muscle area in the short axis of the left ventricle and the length of the apical left ventricle (LVM = (5/6 area × length)). The left ventricular mass index (LVMI) of g/m^2^ was calculated using Devereux’s formula; the cardiac mass was also indicated by lean mass. LVMI was calculated using the Cornell criteria and adjusted for height (measured in meters). The LVEF was determined by summing the diastolic and systolic volumes of the left ventricle using the unidirectional Simpson method: EF = (Dvol − Svol)/Dvol × 100. Diastolic function was assessed using conventional spectral Doppler measurements of mitral inflow and pulmonary venous flow. The E/A ratio, isovolumetric relaxation time (IVRT), and deceleration time of the E wave were also calculated by us. Abnormal RWT and/or LVMI were used to define LVH, as we described in our previous study [35].

### 4.4. Statistical Analysis

Statistical analyses were carried out with the use of SPSS 21.0 software (SPSS, Inc. Chicago, IL, USA). The normality of the data distribution was assessed using the Kolmogorov–Smirnov test. Non-normally distributed parameters were transformed logarithmically. The researcher utilized Student’s *t*-test and ANOVA, as needed, to compare clinical and laboratory parameters. The average standard deviation was utilized to represent information from a Gaussian distribution. Linear regression with the Pearson test was employed to assess connections between continuous variables, while the Spearman correlation test was utilized for categorical variables. The factors that influence endocan and endothelin-1 were investigated using univariate and multivariate linear regression analysis. Values of *p* < 0.05 were considered statistically significant.

## 5. Conclusions

In conclusion, our study confirmed that serum endocan and endothelin-1 alone and combined with NT-proBNP may help to identify IgAN patients at high risk for subclinical heart failure and further vascular disease.

In addition, a possible mechanism for the development of heart failure and vascular injury in CKD patients may be an elevation of central aortic systolic pressure, and therefore the measurement of it could be useful for clinicians. The pathophysiological mechanisms linking the increased prevalence of the biomarker combination with CKD, as well as its association with poor outcomes, must be investigated in larger-scale, multicenter clinical trials that focus on frequent monitoring and exclude medications that disrupt endothelial integrity.

## 6. Limitations of the Study

Certain restrictions must be recognized in our research. First, the present study was a single-center cross-sectional study method with a small number of patients, which could have caused selection bias. Second, we cannot examine the pathological/histological stages of IgAN associations with the measured biomarkers. Thirdly, the renal function was approximated, not physically assessed, despite the widespread acceptance of using eGFR in the literature for characterizing renal function. Fourthly, as a result, the limited occurrence of the biomarker combination from earlier research prevented conducting subgroup analyses in the present study. Moreover, the cross-sectional design of most of the research with the concomitant uncertainty about cause-and-effect associations might limit the strength of the findings. Hence, additional investigations in extensive and separate groups of patients are required to validate these results. Furthermore, the results found could have been affected by potential issues associated with having multiple variables. We did not examine the genetic differences in our studied IgAN patients. Finally, some patients regularly took medications that might have influenced endothelial activity at the time of plasma samples. There were a lack of data about the patient’s medications (ACEIs/ARBs, statins), so we were unable to examine this factor.

## Figures and Tables

**Table 1:** Baseline clinical data.

Clinical Data	All Patients (*n* = 90)	Endocan < 44 ug/L (*n* = 44)	Endocan ≥ 44 ug/L (*n* = 45)	*p*	Endothelin-1 < 231 pg/mL (*n* = 46)	Endothelin-1 ≥ 231 pg/mL (*n* = 44)	*p*	CKD 1–2 (*n* = 42)	CKD 3–5 (*n* = 48)	*p*
Male/female (n, %)	50/40 (56/44)	23/21 (52/48)	26/19 (58/42)	NS	28/18 (61/39)	22/22 (50/50)	NS	25/17 (60/40)	25/23 (52/48)	NS
Age (year)	54.92 ± 14.47	55.9 ± 14.99	53.9 ± 13.85	NS	58.2 ± 12.85	51.4 ± 15.5	0.012 *	49.69 ± 14.80	59.68 ± 12.69	<0.001 *
SBP/DBP (mmHg)	124/78 ± 13/8	122/75 ± 13/10	125/80 ± 13/10	NS	122/76 ± 12/10	125/79 ± 14/10	NS	122/77 ± 14/8	125/78 ± 13/7	NS
MAP (mmHg)	88.91 ± 20.81	86.83 ± 22.49	90.83 ± 22.81	NS	85.41 ± 24.53	94.57 ± 14.3	0.018 *	87.70 ± 23.15	91.91 ± 17.94	NS
HR (beat/min)	65.3 ± 9.49	64.6 ± 9.53	66.2 ± 12.46	NS	67.9 ± 9.86	63.1 ± 9.58	NS	63.8 ± 8.8	66.6 ± 9.87	NS
cfPWV (m/s)	9.9 ± 2.33	11.18 ± 5.72	11.99 ± 7.32	NS	10.7 ± 1.59	10.8 ± 2.77	NS	9.2 ± 2.18	10.6 ± 2.29	0.004 *
Aix (%)	26.5 ± 13.68	38.95 ± 4.87	43.6 ± 3.73	NS	47.33 ± 6.91	39.18 ± 16.21	NS	25.7 ± 15.74	27.9 ± 11.63	NS
Aorta PP (mmHg)	38.4 ± 6.99	36.23 ± 7.35	35.97 ± 9.9	NS	34.5 ± 8.56	36.62 ± 8.77	NS	35.0 ± 3.46	43.5 ± 7.94	NS
SBPao (mmHg)	113.88 ± 12.8	114.55 ± 10.64	121.1 ± 14.03	0.013 *	115.4 ± 15.57	122.1 ± 20.49	0.037 *	115.33 ± 13.01	122.02 ± 12.72	0.044 *
**Biomarkers**										
NT-proBNP (pg/mL)	256.22 ± 404.72	80.5 ± 155.57	148.5 ± 458.54	0.028 *	94.88 ± 167.86	144.85 ± 418.59	0.038 *	173.22 ± 382.22	419.03 ± 775.07	0.035 *
Endocan (ug/L)	59.26 ± 65.75	27.34 ± 7.58	91.18 ± 80.94	n.a.	67.06 ± 84.72	51.45 ± 36.7	NS	42.0 ± 33.5	51.0 ± 37.25	0.047 *
Endothelin-1 (pg/mL)	230.95 ± 101.95	260.96 ± 96.86	221.13 ± 103.03	0.034 *	154.15 ± 47.49	327.94 ± 58.59	n.a.	251.89 ± 108.19	231.58 ± 95.17	NS
**Metabolic Parameters**										
BMI (kg/m^2^)	28.51 ± 5.75	27.93 ± 5.45	29.57 ± 5.95	NS	29.79 ± 5.37	27.25 ± 5.81	0.019 *	27.57 ± 5.21	29.34 ± 6.17	NS
Obesity (n, %)	60 (67)	30 (68)	30 (67)	NS	33 (72)	27 (61)	NS	25 (60)	35 (73)	NS
Hypertension (n, %)	78 (87)	38 (86)	40 (89)	NS	42 (91)	36 (82)	0.040 *	28 (67)	43 (90)	0.005 *
Diabetes mellitus (n, %)	16 (18)	8 (18)	8 (18)	NS	12 (26)	4 (9)	0.013 *	3 (7)	12 (25)	0.013 *
Dyslipidemia (n, %)	32 (36)	14 (32)	17 (38)	NS	17 (37)	14 (32)	NS	16 (38)	16 (33)	NS
Metabolic syndrome (n, %)	39 (43)	17 (39)	22 (49)	NS	21 (46)	18 (41)	NS	16 (38)	23 (48)	NS
**Echocardiographic Parameters**										
LVEF(%)	63.42 ± 5.86	63.96 ± 5.44	62.8 ± 6.18	NS	62.16 ± 5.59	64.7 ± 5.78	0.022 *	65.25 ± 5.39	63.18 ± 6.02	NS
LVMI (g/m^2^)	102.76 ± 27.8	108.05 ± 25.18	105.89 ± 22.14	NS	105.89 ± 23.53	108.05 ± 23.92	NS	95.74.63 ± 22.37	108.94 ± 23.21	0.022 *
LVM (g)	214.68 ± 62.2	222.55 ± 76.42	209.43 ± 47.66	NS	216.87 ± 61.91	211.8 ± 60.03	NS	234.4 ± 75.6	238.0 ± 51.26	NS
RWT	0.43 ± 0.07	0.37 ± 0.04	0.45 ± 0.07	0.026 *	0.43 ± 0.07	0.44 ± 0.08	NS	0.468 ± 0.08	0.472 ± 0.07	NS
LAD (mm)	45.67 ± 6.05	45.56 ± 4.68	45.75 ± 7.13	NS	45.86 ± 6.75	45.17 ± 5.66	NS	44.81 ± 6.25	47.12 ± 5.52	0.014 *
LVEDd (mm)	48.91 ± 4.39	48.71 ± 5.17	49.12 ± 3.4	NS	49.34 ± 4.33	48.5 ± 4.41	NS	48.1 ± 4.37	49.6 ± 4.29	NS
LVESd (mm)	30.17 ± 6.85	30.59 ± 8.74	29.75 ± 4.05	NS	29.86 ± 3.98	30.51 ± 8.98	NS	29.48 ± 8.69	30.81 ± 4.47	NS
IVSd (mm)	11.24 ± 1.44	11.26 ± 1.29	11.25 ± 1.58	NS	11.27 ± 1.41	11.20 ± 1.47	NS	11.04 ± 1.49	11.42 ± 1.37	NS
LVPWd (mm)	11.48 ± 1.57	11.47 ± 1.46	11.51 ± 1.68	NS	11.41 ± 1.46	11.55 ± 1.67	NS	11.29 ± 1.58	11.66 ± 1.54	NS
RVIDd (mm)	25.44 ± 7.56	24.81 ± 5.45	25.2 ± 5.78	NS	24.61 ± 5.67	25.48 ± 5.89	NS	23.52 ± 3.43	27.49 ± 6.23	0.044 *
LVH (n, %)	41 (45)	22 (50)	19 (42)	NS	17 (37)	24 (54)	NS	16 (38)	25 (52)	NS
DD (n/%)	44 (49)	22 (50)	22 (49)	NS	21 (45)	23 (52)	NS	18 (43)	26 (54)	NS
E/A	1.03 ± 0.38	1.01 ± 0.36	1.05 ± 0.39	NS	1.03 ± 0.39	1.0 ± 0.36	NS	1.11 ± 0.39	0.98 ± 0.37	NS
**Laboratory Results**										
Creatinin (umol/L)	155.41 ± 120.24	140.73 ± 102.21	170.09 ± 134.31	NS	164 ± 133.14	147.62 ± 106.64	NS	85.64 ± 15.29	212.77 ± 137.34	n.a.
Urea nitrogen (mmol/L)	14.85 ± 13.56	14.62 ± 11.56	15.01 ± 14.01	NS	14.92 ± 13.76	14.71 ± 12.04	NS	8.1 ± 2.9	17.87 ± 15.41	n.a.
eGFR (mL/min)	47.57 ± 23.24	58.6 ± 26.35	52.79 ± 26.26	NS	53.92 ± 26.24	57.38 ± 26.57	NS	72.61 ± 7.11	34.5 ± 16.32	n.a.
AU (mg/L)	247.62 ± 312.78	218.2 ± 334.19	276.3 ± 287.5	NS	201.36 ± 243.43	292.73 ± 362.37	NS	111.32 ± 204.06	323.63 ± 345.01	<0.001 *
Uric acid (umol/L)	314.23 ± 83.72	302.72 ± 61.55	332.0 ± 92.18	0.045 *	330.92 ± 86.08	298.34 ± 77.05	0.040 *	293.79 ± 61.4	326.38 ± 98.92	NS
Total cholesterol (mmol/L)	4.93 ± 1.31	4.95 ± 1.13	4.92 ± 1.44	NS	4.9 ± 1.48	4.95 ± 1.1	NS	4.98 ± 1.36	4.89 ± 1.3	NS
HDL-C (mmol/L)	1.34 ± 0.42	1.36 ± 0.46	1.32 ± 0.45	NS	1.29 ± 0.4	1.39 ± 0.42	NS	1.42 ± 0.43	1.28 ± 0.41	NS
LDL-C (mmol)	2.90 ± 1.14	2.91 ± 0.98	2.89 ± 1.25	NS	2.93 ± 1.27	2.87 ± 0.97	NS	2.94 ± 1.21	2.85 ± 1.09	NS
TG (mmol)	1.85 ± 1.36	1.63 ± 0.94	2.56 ± 1.62	NS	2.01 ± 1.58	1.69 ± 1.04	NS	1.73 ± 1.47	1.97 ± 1.27	NS
Hb (g/dL)	133.71 ± 28.68	137.52 ± 16.99	138.3 ± 16.1	NS	137.05 ± 17.4	138.8 ± 1566	NS	144.16 ± 14.47	124.88 ± 34.65	0.001 *

* *p* < 0.05 statistical analysis was performed by Student’s *t*-test and ANOVA. SBP: systolic blood pressure; DBP: diastolic blood pressure; MAP: mean arterial pressure; HR: heart rate; cfPWV: carotid-femoral pulse wave velocity; Aix: augmentation index; PP: pulse pressure; SBPao: aorta systolic blood pressure; NT-proBNP: N-terminal pro-hormone of the brain natriuretic peptide; BMI: body mass index; LVEF: left ventricular ejection fraction; LVMI: left ventricular mass index; LVM: left ventricular mass; RWT: relative wall thickness; LAD: left atrial diameter; LVEDd: left ventricular end-diastolic diameter; LVESd: left ventricular end-systolic diameter; IVSd: interventricular septal diameter; LVPWd: left ventricular posterior wall diameter; RVIDd: right ventricular interna dimension at end-diastole; LVH: left ventricular hypertrophy; DD: diastolic dysfunction; E/A: early/late wave of the mitral inflow; eGFR: estimated glomerular filtration rate; AU: urine albuminuria; HDL-C: high density lipoprotein cholesterol; LDL-C: low density lipoprotein cholesterol; TG: triglyceride; Hb: hemoglobin; n.a.: not applicable; NS: not significant.

## Data Availability

The data underlying this article cannot be shared publicly due to Hungarian regulations and the privacy of individuals that participated in the study. The data could be shared on reasonable request to the corresponding author if accepted by the Regional Committee for Medical and Health Research Ethics and local Data Protection Official.

## Supplementary Materials

The supporting information can be downloaded at:https://www.mdpi.com/article/10.3390/ijms251910552/s1.
