# Supplementary material for: The Relationship between Vascular Biomarkers (Serum Endocan and Endothelin-1), NT-proBNP, and Renal Function in Chronic Kidney Disease, IgA Nephropathy: A Cross-Sectional Study
Source: Int J Mol Sci. 2024 Sep 30;25(19):10552. doi: 10.3390/ijms251910552 (PMC11476882; doi:10.3390/ijms251910552)
Supplement: Supplementary file 1 [file ijms-25-10552-s001.zip › ijms-3207587-supplementary.pdf]

Supplementary material: Table S1. Spearman's correlations

	eGFR		LVMI		HDL-cholesterol		SBPao		PWVcf		Hb	
	r	p	r	p	r	p	r	p	r	p	r	p
NT-proBNP	**-0.428**	**<0.001**	**0.354**	**<0.01**	0.110	NS	**0.325**	**<0.01**	**0.469**	**0.034**	**0.242**	**0.001**
Endothelin-1	0.150	NS	0.30	NS	**0.259**	**0.017**	0.89	NS	0.72	NS	0.38	NS
Endocan	-0.35	NS	0.10	NS	-0.60	NS	**0.253**	**0.022**	0.48	NS	0.15	NS

eGFR: estimated glomerular filtration rate; LVMI: left ventricular mass index; HDL: high density lipoprotein; SBPao: aorta systolic blood pressure; PWVcf: carotid-femoralis pulse wave velocity; Hb: hemoglobin; NT-pro-BNP: N-terminal pro-hormone of the brain natriuretic peptide, NS:not significant.

Statistical analysis was performed by Spearman's correlation.

Supplementary material: Table S2. Uni- and multivariate analysis of endocan and endothelin-1

	UNIVARIATE ANALYSIS					MULTIVARIATE ANALYSIS						
Endocan	B	Std. errors	Beta	t	p	B	Std. errors	Beta	t	p	95.0% CI for B lower	95.0% CI for B upper
Gender	13.290	14.057	0.101	0.945	0.347	24.885	18.880	0.174	1.318	0.193	-12.895	62.664
Age	0.276	0.484	0.061	0.569	0.571	0.226	0.798	0.045	0.283	0.778	-1.370	1.822
BMI (kg/m^2)	-1.895	1.223	-0.165	-1.550	0.125	-4.299	1.771	-0.338	-2.428	0.018	-7.842	-0.756
HT	5.552	23.357	0.026	0.238	0.813	-1.283	33.056	-0.005	-0.039	0.969	-67.429	64.862
DM	21.128	18.214	0.124	1.160	0.249	39.832	23.902	0.222	1.666	0.101	-7.996	87.659
Dyslipidemia	-2.979	16.255	-0.020	-0.183	0.855	15.151	20.980	0.097	0.722	0.473	-26.831	57.133
PWVcf (m/s)	0.490	1.108	0.048	0.442	0.659	1.476	1.407	0.146	1.049	0.298	-1.340	4.292
SBPao (mmHg)	0.324	0.338	0.104	0.959	0.340	0.201	0.535	0.056	0.375	0.709	-0.869	1.270
eGFR (ml/min/1m72m2)	88	0.272	-0.035	-0.325	0.746	-0.063	0.465	-0.023	-0.135	0.893	-0.994	0.868
AU (mg/l)	0.002	0.025	0.009	0.084	0.934	-0.001	0.036	-0.006	-0.037	0.970	-0.074	0.071
DD	11.581	19.849	-0.065	-0.583	0.561	-16.416	30.393	-0.086	-0.540	0.591	-77.233	44.401
LVMI (g/m2)	-0.064	0.318	-0.023	-0.203	0.840	-0.372	0.432	-0.126	-0.861	0.393	-1.238	0.493
Endothelin-1												
Gender	-36.502	21.554	-0.0179	-1.693	0.094	-45.939	25.534	-0.219	-1.799	0.077	-97.033	5.156
Age	-1.763	0.728	-0.251	-2.423	0.017	-2.420	1.079	-0.328	-2.243	0.029	-4.579	-0.262
BMI (kg/m^2)	-4.756	1.844	-0.268	-2.578	0.012	-2.475	2.395	-0.132	-1.034	0.305	-7.267	2.316
HT	-83.144	34.946	-0.249	-2.379	0.020	-69.090	44.707	-0.196	-1.545	0.128	-158.549	20.368
DM	-69.315	27.340	-0.264	-2.535	0.013	-47.348	32.326	-0.180	-1.465	0.148	-112.032	17.336
Dyslipidemia	-12.696	25.016	-0.056	-0.508	0.613	-9.691	28.375	-0.042	-0.342	0.734	-66.469	47.087
PWVcf (m/s)	1.136	1.706	0.072	0.666	0.507	0.434	1.903	0.029	0.228	0.820	-3.374	4.243
SBPao (mmHg)	0.300	0.523	0.062	0.573	0.568	0.173	0.723	0.033	0.240	0.811	-1.273	1.620
eGFR (ml/min/1m72m2)	0.580	0.415	0.150	1.398	0.166	-0.003	0.629	-0.001	-0.005	0.996	-1.263	1.256
AU (mg/l)	0.006	0.037	0.017	0.148	0.883	0.035	0.049	0.106	0.712	0.479	-0.063	0.133
DD	0.944	30.218	0.003	0.031	0.975	-39.427	41.105	-0.140	-0.959	0.341	-121.678	42.824
LVMI (g/m^2)	0.085	0.480	0.020	0.176	0.860	0.478	0.585	0.110	0.817	0.417	-0.692	1.648

BMI: body mass index; HT: hypertension; DM: diabetes mellitus; PWVcf: carotid-femoral pulse wave velocity; SBPao: aorta systolic blood pressure; eGFR: estimated glomerular filtration rate; AU: albuminuria; DD: diastolic dysfunction; LVMI: left ventricular mass index. Statistical analysis was performed by uni- and multivariate regression analysis.
